# Information from ecological momentary assessments lead to over-medicalization: Yes

**DOI:** 10.1177/13524585241253077

**Published:** 2024-05-15

**Authors:** Einar A Høgestøl, Pål Berg-Hansen

**Affiliations:** Neuroscience Research Unit, Imaging studies of MS research group, Department of Neurology, Oslo University Hospital & University of Oslo, Oslo, Norway; Institute of Clinical Medicine, University of Oslo, Oslo, Norway/Department of Psychology, University of Oslo, Oslo, Norway; Department of Neurology, Oslo University Hospital, Oslo, Norway

In our contemporary society, we are being increasingly exposed to a spectrum of automated tools designed to capture information about our daily activities, movements, sleep patterns, and more. These tools, commonly referred to as ecological momentary assessment (EMA), involve the repeated collection of real-time data on individuals’ behaviors and experiences in their natural environments.^1^ EMA encompasses methods like ambulatory assessments or exposure sampling, utilizing both manual data collection through questionnaires on paper or smartphone applications and passive retrieval through wearable activity sensors.^2^ One notable advantage of EMA lies in its ecological validity, allowing for generalization to real-life settings. Focusing on the current, momentary state helps reduce the risk of recall bias. The utilization of repeated assessments further provides valuable insight into variations over time.^1^ EMA has proven invaluable in diverse research domains, including studies of psychotic disorders, depression, suicidality assessments, cancer, rehabilitation settings, insomnia, stroke, diabetes, and mental health benefits of birdlife (!), as well as neurological disorders such as stroke, headache, and Parkinson’s disease.^1–4^

In the context of multiple sclerosis (MS), EMA has proven valuable in evaluating neurological symptoms encompassing pain, fatigue, depressive, and cognitive symptoms.^5^ EMA has been utilized to explore the daily temporal associations between physical activity and MS symptoms, offering valuable insights,^6^ and further combining these two above-mentioned approaches.^7^ The concept of medicalization involves interpreting facets of reality, including those expressed in medical terms, and addressing them as medical issues rather than social, political, or existential ones. Over-medicalization occurs when this process yields adverse health effects, such as overdiagnosis, risks associated with increased drug prescriptions, or unnecessary medical procedures. Beyond health implications, over-medicalization can result in negative economic consequences, entailing unnecessary costs for individuals or society, and psychological effects, including stigma or behavioral changes. In addition, it can have detrimental social effects, such as attributing criminal behavior to a personality disorder.^8^ The risk of over-medicalization due to the use of EMA both within and outside a clinical setting in people with MS is, however, less studied.

Outside the context of clinical studies, smartphones and smartwatches have become widely distributed in the population, providing widespread access to instantaneous health-related data, including heart rate, daily step count, and sleep patterns. The constant monitoring facilitated by these innovative wearable health sensors generating EMA data, potentially influencing the stress levels of individuals with MS by serving as a continuous reminder of negative health-related aspects.^9^ For instance, smartwatches assessing sleep quality and providing daily morning feedback may inadvertently contribute to increased stress. The revelation of poor sleep quality, for many individuals, can serve as an exacerbating factor, heightening the perception of its potential negative effects.

One drawback associated with EMA is its potential to impose a greater time burden on patients compared to conventional routine follow-up procedures. The nature of collecting data in real-life settings introduces a challenge concerning the validation of the entered data, particularly in the case of self-reported information.^10^ Given the reliance on patients to accurately report their experiences, there is an inherent risk of subjective interpretation and potential inaccuracies. Unlike in more controlled clinical environments, where data accuracy can be cross-verified, the decentralized nature of EMA may compromise the independent validation of the collected data, raising concerns about the overall reliability of the information acquired. Therefore, while EMA offers valuable insights into the everyday experiences of people with MS, careful consideration must be given to the potential limitations associated with data accuracy and validation procedures and generalizability in real-world settings.

In modern MS care, numerous individuals with MS experience enhanced well-being and reduced symptom burden, due to improved treatment strategies targeting both the underlying disorder and symptomatic treatment. This increasingly large subgroup of people with MS tries to live their lives as close to normal as possible, engaging in work, caring for their families, and actively participating in social activities. However, the integration of EMA into their daily lives may inadvertently serve as a reminder that their state of health deviates from the perceived norm, acting as a constant acknowledgment of their chronic neurological disorder. Also, given EMA’s heightened sensitivity to fluctuations in physical, psychological, and social dimensions, a transient worsening of one or more aspects could potentially trigger unnecessary clinical examinations and imaging acquisition, despite the variation being within the spectrum of normal fluctuations. This emphasizes the need for a balanced consideration of the psychological impact and potential overutilization of healthcare resources when implementing EMA in the routine care of people with MS.

In conclusion, while EMA proves to be a valuable tool in capturing real-time data on the experiences of people with MS, its integration into routine care raises many concerns. The potential overemphasis on minor fluctuations and constant monitoring could lead to increased stress levels and unnecessary use of the healthcare system. Moreover, the decentralized nature of EMA introduces challenges in validating self-reported data, posing concerns about the overall reliability of the information collected. Therefore, careful consideration must be given to the psychological impact and potential overutilization of healthcare resources when incorporating EMA into routine care.
